# Sustainable Synthesis and Dual-Function Sorption of Carbonated Hydroxyapatite for Cadmium and Nitrate Removal

**DOI:** 10.3390/ijms26167766

**Published:** 2025-08-11

**Authors:** Cristina Rodica Dumitrescu, Monica Matei, György Deák, Mădălina Boboc, Elena Holban, Florina Diana Gheorghe

**Affiliations:** National Institute for Research and Development in Environmental Protection, 294 Splaiul Independenței Blv, 060031 Bucharest, Romania; cristina.dumitrescu@incdpm.ro (C.R.D.); dkrcontrol@yahoo.com (G.D.); mada91mada@yahoo.com (M.B.); elena.holban@incdpm.ro (E.H.); diana.dumitru@incdpm.ro (F.D.G.)

**Keywords:** nitrate sorption, heavy metal sorbent, cadmium removal, carbonated hydroxyapatite, wastewater remediation, ion exchange, chemisorption, Rietveld structural refinement

## Abstract

Nitrate (NO_3_^−^) and cadmium (Cd^2+^) are common water pollutants with distinct chemical behaviors, often requiring different removal strategies. This study presents a low-cost synthesis of carbonated hydroxyapatite nanopowder (cHA), Ca_5_(PO_4_)_3-y_(CO_3_)_y_(OH) (*y* = 0.13–0.17), using eggshell waste as a calcium precursor, aimed at removing both NO_3_^−^ and Cd^2+^ from wastewater. SEM and TEM analyses revealed a porous nanostructure with an average particle size of 13.53 ± 6.43 nm and a specific surface area of 7.568 m^2^/g. Adsorption experiments were conducted under varying conditions, including contact time (0.3–3 h), dosage (0.3–2 g/L), initial concentrations (10–100 mg/L for NO_3_^−^; 5–15 mg/L for Cd^2+^), and temperature (22 and 50 ± 2 °C). Cd^2+^ removal reached up to 99% at pH 2–4.5, while NO_3_^−^ removal peaked at 38% in competitive systems, within 30 min. In single-ion systems, maximum nitrate uptake was 19.14 mg/g at 50 °C. Characterization using FT-IR, EDS, and XRD (with Rietveld refinement) confirmed carbonate B-type substitution and structural changes due to ion exchange and chemisorption. The results demonstrate that cHA derived from food waste is an efficient and sustainable sorbent, particularly for cadmium removal in contaminated water.

## 1. Introduction

One of the most pressing global environmental concerns arising from human activities is water pollution caused by heavy metals, dyes, and inorganic compounds such as nitrates and nitrites [1]. These pollutants pose serious risks to both human and animal health [2,3]. Heavy metals such as Pb^2+^, Hg^2+^, Cr^4+^, Ni^2+^, and Cu^2+^, being non-biodegradable and tending to accumulate in living organisms, constitute a major environmental health disorder [4]. Among them, cadmium (Cd^2+^) is particularly dangerous. Numerous studies revealed a wide and complex molecular mechanism induced by the Cd ions presence in physiological fluids, involving DNA and/or protein structure damage and misfolding, exacerbation of inflammatory processes generated by mitochondrial dysfunction and oxidative stress [4,5]. In this context, its toxicity is invasive also due to its chemical similarity to calcium (Ca^2+^), enabling it to form stable complexes with biomolecules and permeate into biological membranes. Therefore, concerning Cd disorders at tissue levels, a lot of systemic diseases that affect immune systems, cardiovascular, reproductive systems, the liver and cerebrovascular central nervous system were reported [6]. Recognized by the International Agency for Research on Cancer (IARC) as a Group 1 carcinogen, cadmium is toxic even at low concentrations, adversely affecting soil microbial activity, plant metabolism, and biological tissues in humans and animals [1]. Similarly, nitrogen-based compounds such as ammonia, nitrite, and nitrate are frequently detected in drinking water, mainly due to agricultural runoff, industrial effluents, and excessive use of household chemicals [7]. High nitrate levels can cause methemoglobinemia (“blue baby syndrome”) in infants and have been linked to colorectal cancer in adults due to prolonged exposure through contaminated water or food preservatives like sodium nitrate [1]. Nitrates are highly soluble, chemically stable, and resistant to conventional water treatment methods, making their removal particularly challenging [8]. Increasingly unsustainable and expensive technologies such as, nanofiltration, electrochemical treatment, reverse osmosis, and biological remediation have been employed [9]. Most of these filtration techniques based on adsorption process that despite advantages (simplicity, efficiency, and selectivity) remained economically unsustainable due to all costs involved in their transformation into affordable, selective, and efficient sorbents, with high adsorption capacity across a broad range of pollutant concentrations and pH levels, as well as fast adsorption kinetics [10]. For instance, for Cd^2+^ and Pb^2+^ removal from wastewater, the maximum adsorption capacity of a synthetic mineral adsorbent prepared by mechanochemical treatments of a mixture containing illite, wollastonite, gypsum, limestone and dolomite powder was between 47.0 and 143.3 mg/g at 25 °C [11]. Also, chitosan-modified phosphate rock powder showed the greatest Cd^2+^ adsorption capacity of 1.88 mg/g, that was 106.59% greater than that of original phosphate rock powder [12]. Among promising materials, apatite minerals—particularly hydroxyapatite (HA)—have gained substantial interest due to their unique physicochemical properties. From this point of view, hydroxyapatite base materials fulfil high expectation concerning thermal and chemical stability in the pH range 5–14 with optimum stability at 10–11, and with decomposition temperature starting with 1000 °C [13,14]. Although HA, a naturally occurring calcium phosphate, is widely used in biomedical applications for its biocompatibility, recently its use in environmental remediation has expanded significantly [15]. It is derived from biological sources such as animal bones, fish scales, and plant materials (e.g., Calendula flowers, papaya leaves, and orange peels) that offered the biocompatibility and low toxicity advantages, and become safe to be used even in water treatment applications [16]. Also, calcite-based sources like clam shells, cockles, and eggshells are used as precursors for hydroxyapatite coprecipitation synthesis [17]. These precursors are abundant, cost-effective, enlarge the potential of synthesis from waste and lend themselves to sustainable HA synthesis. Structural substitutions in HA, such as the incorporation of carbonate (CO_3_^2−^) to form carbonated hydroxyapatite (cHA), can further enhance its functionality. A- and B-type substitutions—replacing OH^−^ or PO_4_^3−^, respectively—induce lattice distortions, reduce crystallinity, and increase the material’s solubility and surface activity [18]. Activated carbons, clays and zeolites and especially polymeric materials (resins) are nowadays conventional adsorbents utilized for heavy metal removal from wastewater, but the enhanced adsorption capacity and ion-exchange ability make cHA a multifunctional material for air, soil, and water pollution control [19], while photoactive composites based on TiO_2_, ZnO, metal-ferrite, etc., should be a solution for organic pollutant degradation (dyes, pesticide, pharmaceutical). Some of them could reach a degradation efficiency more than 93.36% for insecticides, herbicides and fungicides, as was reported in a review paper where a random-effects model was conducted for the meta-analysis [20]. The HA composites with carbon allotropes demonstrated remarkable enhancements in mechanical strength, biocompatibility, and antibacterial effects; however, the synthesis methods are very expensive, still in the emerging stage, and currently limited to small-scale production [21]. Numerous synthesis methods can tailor HA’s properties, especially at the nanoscale that represents an important issue for functional properties Furthermore, the coprecipitation synthesis approaches for hydroxyapatite reduce energy consumption and invasive regents utilization vs. biowaste, which aligns with the concept with green chemistry principles [22,23]. Biocompatibility is one of the very useful characteristics of hydroxyapatite, even outside the medical field, such as in bioremediation. To enhance 2,4,6-trichlorophenol (2,4,6-TCP) degradation in water and soil, extracellular crude enzymes from white rot fungi were immobilized on alginate/hydroxyapatite/chitosan composite microspheres. Compared to free enzymes (68.86% activity), the immobilized form retained 87.2% activity at pH 7 and achieved a 94.7% removal rate under optimal conditions (pH 5, 24 h) [24]. Micro- and nanohydroxyapatite application increased soil pH, reduced available Cu content, and significantly enhanced urease, catalase, and acid phosphatase activities. This also altered the soil bacterial community structure, boosting both abundance and diversity compared to the control [25]. While HA has been extensively studied for heavy metal removal, fewer studies have explored its interaction with nitrate anions, despite some reporting near-complete NO_3_^−^ removal under specific conditions [26,27]. It was also reported a very high removal efficiency of Cu^2+^ (~98%) within the first 15–30 min of contact time, in a single-ion system, using laboratory-synthesized hydroxyapatite nanoparticles. The adsorption mechanism (ion-exchange) operated on the Langmuir model, the copper replacing Ca^2+^ of the hydroxyapatite lattice, which acted as a buffer suspension [28]. Other works have shown high sorption capacities for Sr^2+^, Cd^2+^, Pb^2+^, and Cu^2+^ using HA and its composites [29,30], while modified or thermally treated HA materials have been effective for organic pollutant and pesticide removal through hydrogen bonding and van der Waals interactions [31,32,33,34]. Only, few studies demonstrate the important retention capabilities of hydroxyapatite for nitrate-like anions. Simultaneous nitrogen and phosphorus removal from wastewater was performed using the anammox process coupled with HAP precipitation to create multi-layer granules, which in addition exhibit improved mechanical strength and settleability [35]. At an initial concentration of 100 mg NO_3_^−^/L and pH 3, a hydroxyapatite/alginate nanocomposite achieved 99% nitrate removal efficiency after 240 min of contact at room temperature [36]. Synthetic HA showed a maximum sorption capacity of 93.63 ± 3.25 mg (Sr)/g when exposed to Sr(NO_3_)_2_ solutions ranging from 30 to 100 mg Sr/L in a single-metal system [3]. In the case of cadmium and lead, a three-dimensionally ordered macroporous HA was shown to form a solid solution with Cd^2+^—Ca_10-*x*_Cd_*x*_(PO_4_)_6_(OH)_2_, where *x* = 0.71. Conversely, exposure to Pb^2+^ led to collapse of the macropores due to rapid surface precipitation of pyromorphite, overriding the ion exchange mechanism [37]. Another study using HA powder thermally stabilized at 1000 °C (BET surface area: 36 m^2^/g) found cadmium uptake of 0.34 mol Cd/mol of HA at 10 °C after 120 h. At 50 °C, adsorption increased gradually, reaching 0.55 mol/mol after the same contact time [38]. A HA/chitosan composite exhibited maximum adsorption capacities of 1.9979 mg/g for Cu^2+^ and 0.9965 mg/g for Pb^2+^ at 65 °C, with up to two regeneration cycles [39]. Low-cost HA materials prepared from Tambaqui fish bones showed capacity for adsorbing atrazine (10 mg/L) via van der Waals and hydrogen bonding interactions attributed to collagen residues. The same material removed Co^2+^ at initial concentrations of 2 mg/L (annealed at 600 °C) and up to 30 mg/L (annealed at 900 °C) [40]. Further applications include uranium (VI) removal using a graphene oxide/HA membrane [41], and the development of HA-based hybrid sensors for ultra-trace detection of L-tryptophan and L-tyrosine due to HA’s affinity for organic compounds [42]. For organic pollutant removal, HA demonstrated monolayer adsorption capacities of 4–10 mg/g for phenols at pH 8 and 20 °C, with zinc-doped HA showing higher affinity (15 mg C/g) for catechol compared to undoped HA [43,44]. Additionally, three types of synthetic HA showed pyridine and phenol adsorption capacities (40–46 mg/g) comparable to high surface area activated carbons derived from coconut shell and rubber seed coat [45].

Recent studies have extensively examined the competitive adsorption of cations in multi-cation systems on hydroxyapatite-based composite, the simultaneous adsorption of both anions and cations, like nitrates—a condition commonly encountered in real wastewater scenarios—remains largely unexplored.

Given the increasing demand for sustainable and cost-effective solutions to remove multiple contaminants from wastewater, this study aims to develop a multifunctional adsorbent only made of carbonated hydroxyapatite (cHA) nanopowder, for the simultaneous removal of cadmium (Cd^2+^) and nitrate (NO_3_^−^) ions, in dynamic contact condition of sorbent and polluted solutions. To this end, a simple, low-cost, and energy-efficient synthesis method was employed, utilizing recycled eggshells as a calcium source and phosphate precursors, without the need for thermal stabilization. This study investigates both competitive adsorption from Cd(NO_3_)_2_ solutions under highly acidic conditions (2% HNO_3_) and single- ion system (KNO_3_, at pH 5.5–7), mimicking real-world wastewater scenarios. In addition to quantifying residual ion concentrations in treated solutions, comprehensive analyses of the structural, morphological, and surface chemical modifications of cHA and a complex solid solution formation post-adsorption (Ca_10-*x*_Cd_*x*_(PO_4_)_6-*y*_(CO_3_)_*y*_(OH)_2_) are conducted. By targeting the concurrent removal of chemically dissimilar ions, this research highlights the potential of cHA as a low-impact, sustainable sorbent for multifunctional water treatment applications.

## 2. Results

### 2.1. Compositional and Morpho-Structural Characterization of Hydroxyapatite as Sorbent

**Figure 1:**
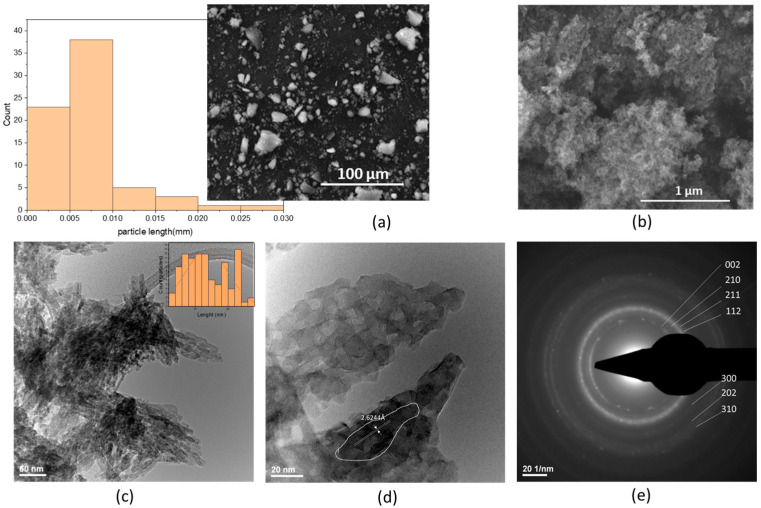
Hydroxyapatite powder electronic microscopy images: SEM micrographs (**a**) 2000× and particle length distribution and (**b**) 100,000×, TEM micrographs (**c**,**d**), and (**e**) SAED pattern.

**Scanning Electron Microscopy** (SEM) images (Figure 1a,b) reveal poorly defined intergranular boundaries, with nanometric particles aggregated into larger, porous clusters exhibiting globular morphology and low structural coherence. At a magnification of 2000×, the HA grains display rounded edges—indicative of reduced crystallinity and a strong tendency towards nanoparticle agglomeration. The particle dimensions range from less than 2 μm to a maximum of approximately 27 μm, with an average size of 6.774 ± 4.657 μm, as shown in the particle size distribution (Figure 1a). At higher magnification (100,000×) (Figure 1b), the aggregates exhibit a highly porous, aerated texture composed of irregularly shaped nanometric particles. Transmission Electron Microscopy (TEM) analysis (Figure 1c) further confirms a plate-like morphology of nanoscale HA crystallites, preferentially oriented along the c-axis. The particles range in size from 2.45 nm to 27.66 nm, with an average length of 13.529 ± 6.429 nm, consistent with the hexagonal crystal structure typical of hydroxyapatite formed under ambient conditions. At 20 nm scale (Figure 1d), overlapping plate-like particles with sizes below 20 nm can be clearly observed. A well-defined crystallite with a length of 60.902 nm, width between 7.614 nm and 11.852 nm, a surface area of 825.18 nm^2^ was identified, corresponding to the [002] Miller plane, with an interplanar spacing (d-spacing) of 2.6244 Å. Selected Area Electron Diffraction (SAED) patterns (Figure 1e) confirm the polycrystalline nature of the sample, with diffraction spots corresponding to the [211], [300], [310] and [202] crystallographic planes [7].

**Figure 2:**
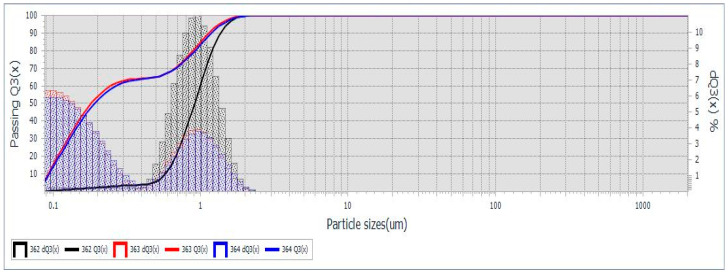
cHA powder granularity measured by Laser Particle Sizer, Mie theory, triplicate.

**Grain size distribution** of the synthesized cHA powder was assessed using optical particle sizing in the wet mode, based on Mie scattering theory. SEM and TEM analyses confirmed nanometric particle formation via coprecipitation, with no significant particle growth during maturation (Figure 1b–d). However, drying at 100 °C in the presence of ammonium hydroxide led to agglomeration into submicron- and micron-sized clusters (Figure 1a). Particle size profiles (Figure 2) revealed bimodal distributions for samples 363 and 364, with ~65% of particles < 259 nm and the remainder approximately 1 μm (D10 = 0.092 μm, D50 = 0.195 μm, D90 = 1.161 μm). Sample 362 exhibited a monomodal distribution (D50 = 0.876 μm) (Supplementary Materials Table S1). Based on hydroxyapatite’s theoretical density (3.16 g/cm^3^) and MaS control 3x software for statistical analysis, specific surface areas were 99,270–102,417 cm^2^/g for samples 363/364, and 25,354 cm^2^/g for sample 362. In addition, the use of the wet method for analysis allows for better dispersion of the granules in suspension, but it also promotes segregation. This explains the differences between sample 362 and the pair 363–364. An average specific surface area of 7.6 m^2^/g is not representative for cHA powder synthesized by coprecipitation, but explainable for a very short grinding final operation (5 min). Also, the optical measurement method enabled generating accurate statistical grain size distribution, a mean diameter size, etc., but cannot detect the surface and internal porosity of aggregates, observed in SEM images (Figure 1b), which may contribute to an even greater effective surface area relevant for adsorption processes.

**Figure 3:**
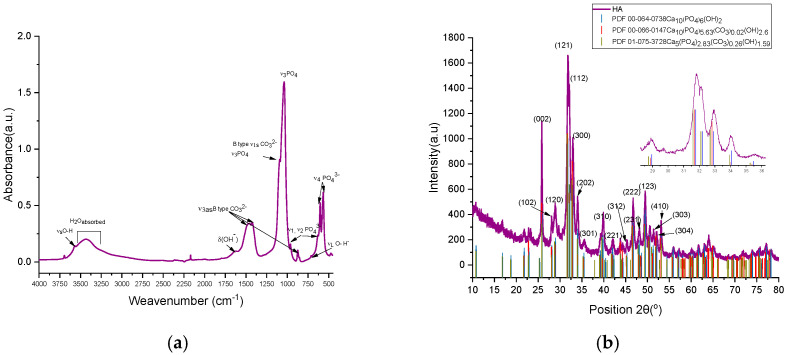
(**a**) FTIR spectra of cHA; (**b**) X-ray diffraction plot for the cHA hydroxyapatite powder sample.

**The FTIR absorption spectra** for the hydroxyapatite powder sample showed the bending and stretching vibrations identified for the PO_4_^3−^ and (CO_3_)^2−^ groups characteristic of carbonated hydroxyapatite as shown Figure 3a [19]. A low-intensity absorption band in the range 1950–2100 cm^−1^ may be associated with the combination *ν_3_* and *ν_1_* vibrations (PO_4_) [46,47,48]. Trigonal planar symmetry D3h characteristics of CO_3_^2−^ functional groups generated IR active vibrations: out-of-plane bending vibration *ν_2_*, *ν_1_* and *ν_3_*, that appeared close to PO_4_ group at 872 cm^−1^ (*ν_2_*), as a shoulder a symmetrical vibration at 1096 cm^−1^ and also asymmetrical vibrations at 1420 and 1453 cm^−1^, all four assigned to the stretching/tensile vibration of the (CO_3_)^2−^ that substituted the phosphate groups (PO_4_)^3−^ of the hydroxyapatite lattice (type B substitution) [49]. The substitution type B of the carbonate hydroxyapatite powder sample was confirmed by the presence of stretching vibrations of O-H bond at 631 and 3571 cm^−1^ and bending vibration at 1643 cm^−1^, where the oxygen is bounded with P of the PO_4_ tetrahedra characteristic to hydroxyapatite crystal structure. The absorbed water was identified by the broad adsorption band between 3681 and 3000 cm^−1^ that was typical to the powder samples prepared with KBr base, being a hygroscopic material [46,47,48].

The degree of type B substitution of (PO_4_)^3−^ by (CO_3_)^2−^ was determined empirically using OriginPro 2021 software for an accurate background and absorbance amplitude measurement [50]. Therefore, for the content of carbonate groups involved in B-type substitution, as the rate between the heights peaks of the wavenumber 1423 and 1039 cm^−1^ (Equations (9) and (10)), A_1423_/A_1039_ was 0.2130 and A_875_/A_1039_ = 0.058, R_avg_ = 0.1357; therefore, it could be considered a relative substitution index *y* of solid solution Ca_5_(PO_4_)_2.87_(CO_3_)_0.13_(OH).

The compositional and crystalline phase characteristics of carbonated hydroxyapatite (cHA) powder samples (Figure 3b) were investigated via **X-ray diffraction (XRD)**, with reference to standard patterns PDF 00-064-0738 (blue), PDF 00-066-0147 (red), and PDF 01-075-3728 (olive), corresponding to hydroxyapatite (HA) and carbonated HA (cHA) with hexagonal symmetry (P6_3_/m space group). All characteristic reflections of hexagonal HA were observed in the cHA samples, with no secondary calcium phosphate phases detected. The XRD pattern of the cHA sample exhibited partial alignment with carbonated HA reference patterns (C:P ratios *y* = 0.02 and 0.26), showing systematic shifts in *2θ* relative to stoichiometric HA (PDF 00-064-0738) and especially to cHA diffraction pattern (purple) (Figure 3b). Peak shifts suggest incorporation of carbonate groups into the phosphate lattice sites (B-type substitution), as illustrated in the inset of Figure 3b insert.

The cHA powder samples were structurally refined using two reference models: PDF 00-064-0738 and PDF 01-075-3728, which showed the best match in terms of 2*θ* diffraction peak positions compared to the experimental data (Table 1). When Rietveld structural refinement was performed against both reference patterns simultaneously, the resulting phase composition consisted of 59.2% stoichiometric hydroxyapatite (PDF 00-064-0738) and 40.8% carbonated hydroxyapatite (PDF 01-075-3728). The carbonated hydroxyapatite phase exhibited significant distortions in its crystal lattice relative to the reference, leading to an 18.31% increase in unit cell volume and a reduced structural density of 2.58 g/cm^3^ (compared to the reference value of 3.17 g/cm^3^). The refined chemical formula was determined to be Ca_5_P_2.83_C_0.26_O_13.69_ compared to the reference formula Ca_5_P_2.829_C_0.26_O_13.686_H_1.59_. In contrast, the stoichiometric hydroxyapatite phase showed only a subtle decrease in unit cell volume (0.001%) compared to the reference, yet this small structural change was associated with a different refined formula: Ca_5_P_3_O_12.93_H_0.16_, as opposed to the ideal Ca_5_P_3_O_13_H_1_. In this case, a subunitary Goodness of Fit (GOF) value may suggest overfitting, poor error estimation, or incorrect background correction (FDS to ADS)—particularly when using “.raw” format data that require conversion prior to refinement.

However, agreement indices such as R_exp_ and R_p_ below 10%, along with a GOF close to 1, indicate a good match between the experimental and calculated diffraction patterns-when refinement was performed using only the carbonated hydroxyapatite phase Ca_5_P_2.829_C_0.26_O_13.686_H_1.59_. As a result of the Rietveld structural refinement, the P1 occupancy was confirmed at 0.943 and using Equations (7) and (8), the degree of substitution (*y*) of CO_3_^2−^ for PO_4_^3−^ was calculated, values that are consistent with those estimated from FTIR spectral data and yielding a refined formula of Ca_5_P_2.829_C_0.171_O_13.69_H_2_. This phase is characterized by a lower structural density (2.59 g/cm^3^), microstrain of 0.165%, and an average crystallite size of 1.45 ± 0.055 nm. The much smaller crystallite size in cHA is due to carbonate-induced lattice microstrain and defects, which prevent large crystal domain growth. In contrast, pure hydroxyapatite forms larger, higher crystallinity (58.28%), well-ordered crystallites under the same or similar synthesis conditions due to lack of structural distortion and lower defect density.

### 2.2. Hydroxyapatite Powder Adsorption Capacity Characterization

#### 2.2.1. Supernatant Liquid Analysis After cHA Powder Contacted with Aqueous Solutions of Cd (NO_3_)_2_ and KNO_3_

**Figure 4:**
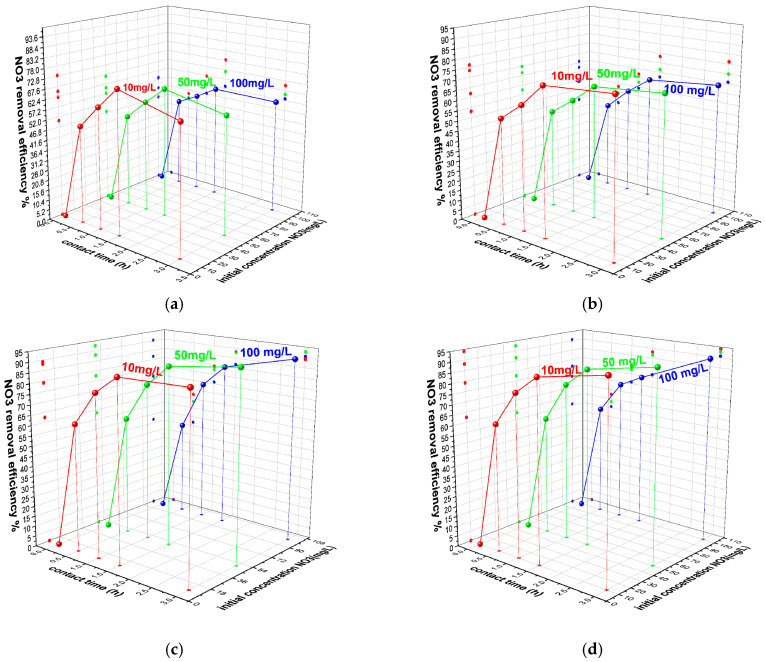
The variation in sorbent removal efficiency of nitrate ions versus contact time at 25 ± 2 °C and initial concentration (**a**) for 0.3 g of cHA, (**b**) 0.5 g cHA, (**c**) 1 g cHA and (**d**) 2 g cHA with an initial concentration of 10 mg NO_3_/L, 50 mg NO_3_/L and 100 mg NO_3_/L.

Potassium nitrate solutions (10, 50, and 100 mg NO_3_^−^/L) were contacted, in dynamic conditions, with 0.3, 0.5, 1, and 2 g of cHA powder for 0.5, 1, 1.5, and 3 h at 25 ± 2 °C and 50 ± 2 °C. Residual nitrate concentrations were determined by UV–Vis spectroscopy, and the removal efficiency (E%) and adsorption capacity (Q_e_, mg/g) were calculated using standard equations (Equations (11) and (12)). As shown in Figure 4a–d, over 60% of nitrate was removed within the first 60 min in all 12 experimental series. The lowest initial efficiency (62%) was recorded for 10 mg/L NO_3_^−^ with 0.3 g of cHA, while the highest (79.9%) occurred with 2 g under the same conditions. Adsorption continued to increase gradually beyond 90 min—for instance, from 62% to 67% for the 0.3 g/10 mg L^−1^ sample. After 3 h, the highest efficiency (95%) was achieved for the HA10N sample using 2 g of adsorbent and 10 mg/L nitrate. These results confirm cHA’s high and dose-dependent nitrate removal performance under dynamic adsorption conditions.

**Figure 5:**
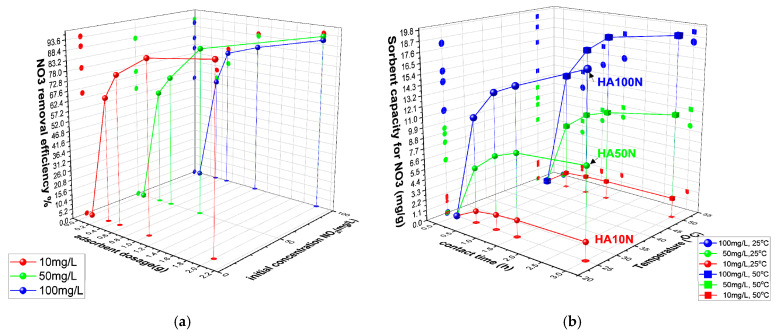
The variation in (**a**) cHA sorbent efficiency (%) for nitrate ions versus sorbent dosage and initial concentration (25 ± 2 °C); (**b**) sorbent capacity versus contact time and temperature (25 ± 2 °C and 50 ± 2 °C) for an initial concentration of 10–100 mg NO_3_/L.

As shown in Figure 5a, nitrate removal efficiency (E%) increased with cHA dosage, reaching 94.08%, 92.6%, and 90.58% for initial nitrate concentrations of 10, 50, and 100 mg/L, respectively, after 3 h. While E slightly decreased with higher nitrate concentrations, overall performance remained high. Sorption capacity (Q_e_) increased with contact time (0.5–3 h), especially for 100 mg/L solutions, reaching 18.11 mg/g at 25 °C and 19.16 mg/g at 50 °C (Figure 5b).

This modest thermal gain (~5.5%) suggests limited benefit from heating of the entire system during the adsorption process. In the first 30 min, 2 g of cHA removed 61–68% of nitrate from 100 mg/L solutions (11.14–12.96 mg/g), after which the adsorption rate slowed to <3 mg/g per half hour. Across all concentrations, >75% removal was observed at 20 °C, increasing with temperature, particularly for lower-concentration solutions—up to ~90% for 10 mg/L—indicating an endothermic adsorption process. As near-maximum efficiency was reached within 3 h, longer contact times may be economically inefficient. This aligns with previous reports indicating that the optimal pH for nitrate adsorption is approximately 6 [51].

**Figure 6:**
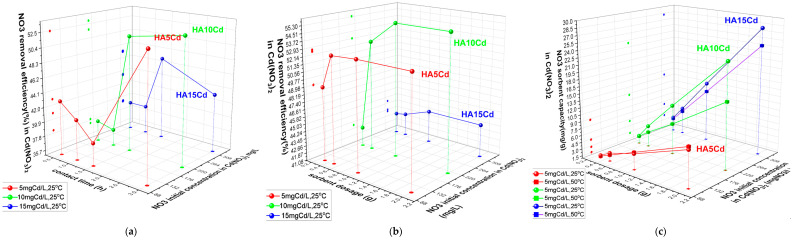
The variation in (**a**) nitrate sorbent efficiency (%) of 0.5 g cHA versus contact time and initial concentration (25 ± 2 °C); (**b**) NO_3_ removal efficiency (%) of cHA sorbent vs. sorbent dosage (g) and initial concentration of NO_3_ in Cd(NO_3_)_2_ (25 ± 2 °C), after 3 h contact time; (**c**) sorbent capacity (mgNO_3_/g) versus sorbent dosage and temperature (25 ± 2 °C and 50 ± 2 °C) for an initial concentration of NO_3_ in Cd(NO_3_)_2_ 5–15 mg Cd/L.

The **adsorption** behavior of cHA towards **nitrate** in Cd(NO_3_)_2_ + 2%HNO_3_ solutions (5–15 mg Cd/L; 105.5–316.6 mg NO_3_^−^/L) was studied under acidic conditions (initial pH 0.5–3) due to 2% HNO_3_ content (Figure 6a–c). In contrast, cHA suspensions initially exhibited pH ≈ 8, which rose to 5–6 after 3 h of contact. Using 0.5 g of cHA at 25 ± 2 °C, nitrate removal efficiencies after 3 h were 52.5% (HA5Cd), 51.6% (HA10Cd), and 47.2% (HA15Cd), with the highest removal observed within the first 90 min. Initial adsorption was lower at higher Cd(NO_3_)_2_ concentrations due to greater proton competition and Ca^2+^ leaching. pH increase during contact promoted OH^−^ formation (as Ca(OH)_2_), influencing competitive sorption dynamics. After 3 h, pH stabilized at ~6, supporting efficient NO_3_^−^ removal, in line with literature indicating pH ≈ 6 as optimal. Varying adsorbent dosages (0.5–2 g) showed that 1 g yielded peak efficiencies—up to 55.4% for 211 mg NO_3_^−^/L—with minimal improvements beyond this dosage. Maximum sorption capacity (Q_e_) reached 28 mg NO_3_^−^/g for 2 g cHA at 25 ± 2 °C, particularly in high Cd(NO_3_)_2_ concentration systems (316.6 mg NO_3_^−^/L), demonstrating effective nitrate uptake under competition. Lower Q_e_ values were recorded at 50 ± 2 °C, confirming the exothermic nature of the adsorption process.

**Figure 7:**
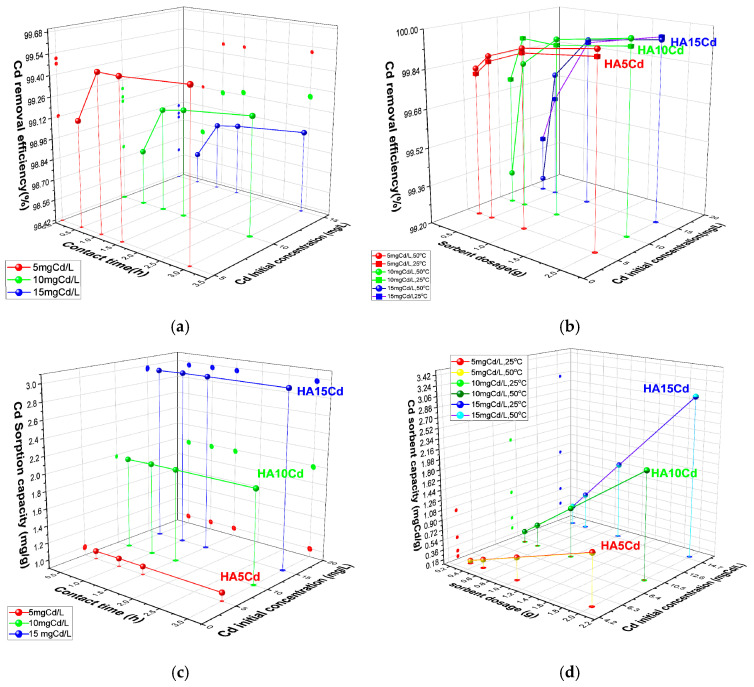
The variation in (**a**) removal efficiency (%) of 0.5 g cHA powder for Cd^2+^ ions versus contact time and initial concentration (25 ± 2 °C); (**b**) Cd removal efficiency (%) versus contact time and initial concentration mgCd/L (25 ± 2 °C); (**c**) sorption capacity (mg/g) for Cd versus contact time and initial concentration mgCd/L (25 ± 2 °C); (**d**) sorption capacity (mg/g) for Cd versus powder dosage and initial concentration mgCd/L (25 ± 2 °C and 50 ± 2 °C).

The **cadmium removal efficiency** (E, %) of 0.5 g cHA powder samples was evaluated after exposure to cadmium nitrate aqueous solutions with initial Cd^2+^ concentrations of 5, 10, and 15 mg/L (Figure 7a). Compared to adsorption from KNO_3_ solutions, the presence of Cd(NO_3_)_2_ induces competitive adsorption between Cd^2+^ and NO_3_^−^ ions on the active sites of the porous cHA nanoparticles. As a result, cadmium removal kinetics are rapid within the first 30 min, with the highest removal efficiency observed for the lowest initial concentration (5 mg/L), reaching 99.12%. Removal efficiency decreases slightly at higher Cd concentrations (15 mg/L). During the subsequent 30 min, removal efficiency continues to increase by approximately 1%, after which it stabilizes and remains nearly constant over the remaining contact period. The cadmium sorption capacity (Q_e_) of the cHA powder exhibits a similar temporal trend, with more than 98% of the initial Cd amount removed within the first 30 min (Figure 7b). For a fixed adsorbent dosage of 0.5 g, Q_e_ increases with initial Cd concentration, ranging from approximately 1 mg/g at 5 mg/L to 2.9 mg/g at 15 mg/L (Figure 7c). Regarding the influence of temperature on sorbent capacity (Figure 7d), it can be observed that the Q_e_ values obtained at 25 ± 2 °C are almost identical to those at 50 ± 2 °C.

#### 2.2.2. Adsorbent Powder Analysis After Contact with Aqueous Solutions of NO_3_^−^ and Cd^2+^

The cHA powder samples after 3 h of contact with KNO_3_ and Cd(NO_3_)_2_ different concentration solutions, with the best results of efficiency and adsorption capacity, were analysed by X-ray diffraction, FTIR spectroscopy and EDS in order to highlight the retention mechanisms of the two pollutants Cd^2+^ and NO_3_ ions. The denomination of powder samples and the concentrations of the solutions to which they have been exposed is presented in the Table 2. 

**Figure 8:**
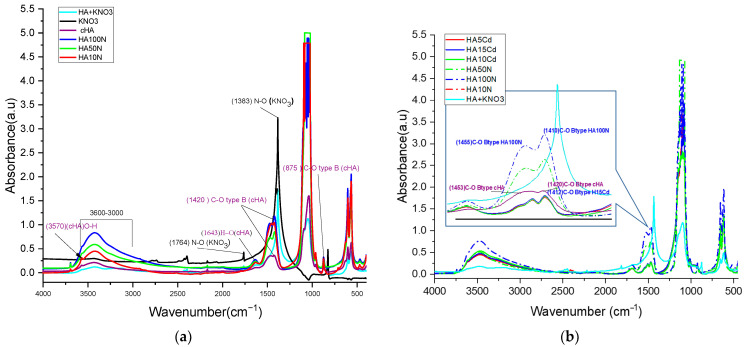
Comparative FTIR spectra for: (**a**) HA10N, HA50N, and HA100N after 3 h contact time of cHA powder with KNO_3_ aqueous solutions 10, 50 and 100 mg NO_3_/L and (**b**) HA10N, HA50N, HA 100N and HA5Cd, HA10Cd, HA15Cd, after 3 h contact time of cHA powder with Cd(NO_3_)_2_ + 2%HNO_3_ aqueous solutions 5, 10 and 15 mg Cd/L.

**FTIR spectra analysis** were determined on HA10N, HA50N, and HA100N samples to evaluate structural changes on crystal lattice due to adsorption process Figure 8a. In this respect, the highest NO_3_^−^ retention capacities were observed for all samples after 180 min of contact. Characteristic PO_4_^3−^ vibrational bands of hydroxyapatite were identified at HA10N, HA50N, and HA100N samples: the *ν_3_* asymmetric stretch (shifted to ~1098 cm^−1^), strong bands at 1035, 597, and 560 cm^−1^ (shifting to 1039–1041, 604, and 577 cm^−1^, respectively), and the *ν*_1_ symmetric stretch at 964 cm^−1^. The CO_3_^2−^ group showed shifts in the *ν*_3_ band from 1456 to 1452 cm^−1^, *ν*_2_ from 880 to 875 cm^−1^, and *ν*_4_ from 712 to 713 cm^−1^ [36]. Notably, the *ν*_3_ (CO_3_^2−^) band shifted from 1423 to 1418 cm^−1^ in HA100N, with increased amplitude. Smaller shifts and moderate amplitude increases were noted for HA50N and HA10N, suggesting a concentration-dependent interaction. The band at ~1420 cm^−1^ increased in intensity across all treated samples, attributed to vibrational coupling between C–O in CO_3_^2−^ and N–O stretching from NO_3_^−^ (1383 cm^−1^). Additional NO_3_^−^-related bands at 1383 and 825 cm^−1^ overlapped with carbonate bands (1420 and 880 cm^−1^), indicating NO_3_^−^ incorporation via surface complexation and not phosphate group substitution [52]. The absence of the 1760 cm^−1^ N–O vibration and the retention of OH-related bands at 631, 3571, and 1643 cm^−1^ further confirmed that NO_3_^−^ did not replace lattice OH^−^ groups [53].

These findings support an adsorption mechanism where NO_3_^−^ compensate electrostatically the negative charge sites deficit generated by B-type PO_4_^3−^ → CO_3_^2−^ substitution, likely forming N–O–C-type bond. The increasing peak intensity at ~1420 cm^−1^ correlates with NO_3_^−^ concentration (HA100N > HA50N > HA10N), confirming its progressive adsorption. Comparing KNO_3_ and Cd(NO_3_)_2_-treated samples (HA100N–HA10N and HA15Cd–HA5Cd), the increased amplitude of bands at 1420, 1453, and 875 cm^−1^—typical of B-type CO_3_^2−^—suggests preferred NO_3_^−^ adsorption at these sites next to the carbonate group (Figure 8b). Resonance at ~1410 cm^−1^, involving C–O (1420 cm^−1^) and N–O (1383 cm^−1^), along with coupling at 875 (C–O) and 824 (N–O) cm^−1^, further supports this interaction. As shown in Supplementary Materials Table S2, KNO_3_-treated samples exhibited stronger C–O vibrational modes than either cHA before or after Cd(NO_3_)_2_-exposer samples, confirming higher NO_3_^−^ adsorption.

**Figure 9:**
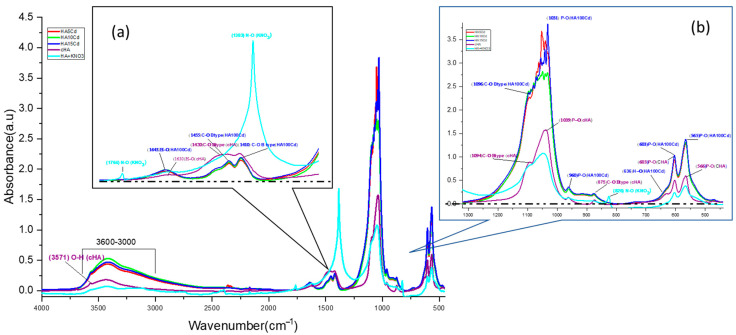
Comparative FTIR spectra for the HA sample before contact (purple), KNO_3_ + HA powder, and cHA after different contact time with aqueous solutions 5, 10 and 15 mg Cd/L (HA5Cd, HA10Cd and HA15Cd) (**a**) detail of spectra for range 2000–1000 cm^−1^ (**b**) detail of spectra for range 1300–500 cm^−1^.

**FTIR spectral analysis of the HA5Cd, HA10Cd, and HA15Cd** samples—each exhibiting Cd^2+^ sorption efficiencies between 98.98% and 99.54%—was performed to qualitatively and semi-quantitatively assess the competitive adsorption behavior of NO_3_^−^ and Cd^2+^ ions from cadmium nitrate solutions (5, 10, and 15 mg/L) (Figure 9a). The FTIR spectra of the three samples showed nearly identical overlap, with distinguishable differences compared to the pristine cHA spectrum, indicating structural alterations in the cHA lattice following exposure to Cd(NO_3_)_2_. These changes are attributed primarily to cationic substitution (Cd^2+^→Ca^2+^) and secondarily to NO_3_^−^ addition adjacent to B-type CO_3_^2−^ groups. The cationic exchange led to a marked increase in both intensity and broadening of the characteristic ν_4_ PO_4_^3−^ vibration at ~1040 cm^−1^. This band, originally at 1.56 a.u. in cHA, increased to 3.64, 3.52, and 2.72 a.u. in HA15Cd, HA10Cd, and HA5Cd (Supplementary Materials Table S2), respectively, due to coupling with the strong Cd–O bond vibration at 1047 cm^−1^ (Figure 9b) [52]. Furthermore, a new absorption band at 542 cm^−1^, attributed to Cd–O vibrations within a cadmium oxide-like environment [53], appeared following Cd^2+^ → Ca^2+^ substitution. This band contributed to enhanced intensity of the PO_4_^3−^ vibrations at 604 and 564 cm^−1^ through the formation of P–O–Cd linkages.

Significant enhancement was also observed in the absorption bands at 1635 cm^−1^ and within the 3600–3000 cm^−1^ range, centered at 3419 cm^−1^, in the Cd-exposed samples relative to pristine cHA. These enhancements (Supplementary Materials Table S2) are ascribed to synergistic vibrational coupling: OH stretching within the HA lattice, N–O bond vibrations from incorporated NO_3_^−^, structural water, and the strengthened hydrogen bonding in Cd–O–H units compared to Ca2–O–H in pristine HA [54]. The increased amplitude in the 3600–3000 cm^−1^ region (Supplementary Materials Table S2) is likely due to OH^−^ groups mobilized during partial Ca^2+^ dissolution from the HA lattice in acidic Cd(NO_3_)_2_ solution, subsequently adsorbed onto active surface sites. Additionally, peaks originally located at 1423 and 1456 cm^−1^ in cHA shifted to lower wavenumbers (1411 and 1450 cm^−1^, respectively), trending towards the characteristic N–O stretching frequency of NO_3_^−^ at 1383 cm^−1^, further confirming nitrate incorporation into the lattice.

**Figure 10:**
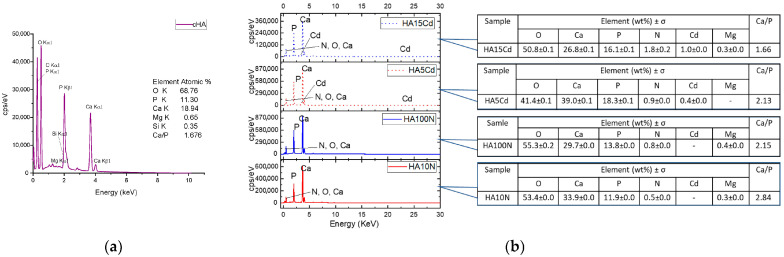
Elemental composition of powder samples determined by EDS: (**a**) cHA and (**b**) HA10N, HA100N, HA5Cd and HA15Cd.

The **Energy Dispersive Spectra** (EDS) reveal the presence of Ca, P, O in a stoichiometric rate Ca/P = 1.676 (Figure 10a). However, the Ca/P rate range between 1.20 and 2.0 has been reported for carbonated hydroxyapatite [26,55,56], the reason for the deviation from the stoichiometry compared with pure HA, in addition to native carbonate type B substitution, being the amounts of elements like Si, which are involved in anionic substitutions (e.g., PO_4_ with SiO_4_), as well as cations replacement of Ca^2+^ positions with Mg^2+^, Na^+^, etc. [18,57]. It is observed that the cHA powder does not contains Cd ion before exposure to polluted water. The cHA powder samples elemental composition after 3 h contact with simulated polluted water (Figure 10b) showed a nonstoichiometric tendency for enrichment in calcium content, even at samples exposed to concentrated cadmium nitrate solutions (HA5Cd and HA15Cd). The cadmium content of these two samples increased with the increase in the concentration of the cadmium nitrate solution: 1 wt% at initial concentration 15 mgCd/L and 0.4 wt% for 5 mgCd/L, respectively. In the same manner, the nitrogen content of all samples increased with an initial NO_3_ concentration of solutions, respectively, from 1.8 ± 0.2 wt% for 316.6 mg NO_3_/L (HA15Cd), to 0.5 ± 0.0 wt% for 10 mg NO_3_/L.

**Figure 11:**
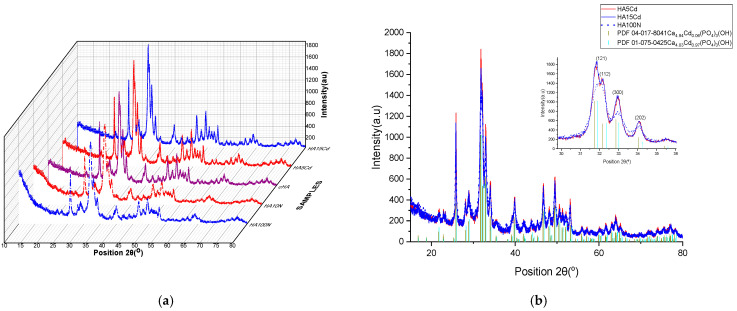
XRD plots (**a**) comparative patterns of HA10N, HA100N, cHA, HA5Cd and HA15Cd samples; (**b**) overlapped patterns of HA5Cd, HA15Cd and HA100N samples.

The **comparative X-ray diffraction (XRD) analysis** of cHA samples before and after exposure for 3 h to aqueous solutions of KNO_3_ (at concentrations of 10 and 100 mg NO_3_^−^/L, HA10N and HA100N) and Cd(NO_3_)_2_ (at concentrations of 5 and 15 mg Cd/L, HA5Cd and HA15Cd) is presented in Figure 11a. The similarity in the diffraction patterns across all five samples indicates that the cHA crystal lattice remains intact after 3 h of adsorption, suggesting that the material can continue to be used until the saturation of adsorption sites, without structural decomposition.

Subtle peak broadening observed in the HA10N and HA100N diffraction patterns correlates with the amount of physiosorbed nitrate (4.4 and 14.1 mg NO_3_^−^/g HA, respectively). In contrast, for HA5Cd and HA15Cd—despite the low levels of adsorbed cadmium (0.0089 and 0.031 moles Cd per 0.996 moles HA)—the diffraction peaks are sharper compared to the unexposed cHA sample. These changes suggest cadmium incorporation into the HA structure and are more clearly evidenced when compared with reference patterns of the solid solutions Ca_4.94_Cd_0.06_(PO_4_)_3_(OH) and Ca_4_Cd(PO_4_)_3_(OH), as shown in Figure 11b. As illustrated in Figure 11b inset, the *2θ* diffraction angles of HA5Cd and HA15Cd closely match the pattern of the solid solution with full Ca^2+^/Cd^2+^ substitution (*x* = 0.97, PDF 04-016-8842), rather than the partial substitution pattern (*x* = 0.06, PDF 04-017-8041), indicating a high degree of Ca/Cd substitution. The structural changes induced in cHA after 3 h of exposure to KNO_3_ and Cd(NO_3_)_2_ were further elucidated via Rietveld refinement, using the aforementioned reference patterns. For HA10N and HA100N, refinement was based on PDF 00-066-0147 (hexagonal hydroxyapatite) and PDF 00-064-0738 (carbonate hydroxyapatite, Ca_10_(PO_4_)_5.63_(CO_3_)_0.02_(OH)_2_). In these cases, modifications in the lattice could be attributed to the incorporation of NO_3_^−^ groups near B-type CO_3_^2−^ sites. However, in the ICDD PDF5+ database, no XRD references related to the substitution of NO_3_ to the structure of hydroxyapatite were found.

Structural refinement by the Rietveld method for the HA10N, HA100N, HA5Cd, and HA15Cd samples was performed using relevant reference patterns from the ICDD PDF-5+ database, as presented in Table 3. For the HA10N and HA100N samples, due to the absence of powder diffraction files corresponding specifically to nitrate incorporation into the hydroxyapatite structure, the refinement was carried out using PDF 01-075-3728 (carbonated hydroxyapatite) and PDF 00-064-0738 (stoichiometric hydroxyapatite) as structural models, selected to highlight potential dimensional modifications of the hydroxyapatite unit cell (cHA) following nitrate adsorption from the two solutions with concentrations of 10 and 100 mg NO_3_^−^/L, respectively. Adsorption of nitrate on cHA enhance lattice strain already present due to carbonate substitution such as slight shifts in peak positions, indicating small changes in unit cell parameters and also increased peak broadening (Figure 11b insert picture), reflecting enhanced microstrain (0.165% at cHA, 0.624 for HA10N and 0.750% at HA100N) or reduced crystallinity from 57.7% at cHA, 50.49% HA10N and 48.14% for HA100N, respectively. These effects are attributed to surface disorder and local lattice distortion rather than deep substitution into the CHA crystal structure [58].

The structural refinement of the cadmium-loaded hydroxyapatite samples (HA5Cd and HA15Cd) yielded the best fitting results using PDF 04-017-8041 (*x* = 0.06) and PDF 01-075-0425 (*x* = 0.97) as structural models, respectively. Based on the Rietveld refinement of the HA5Cd sample and considering a calcium site occupancy of Ca(II) = 0.9817, the resulting chemical formula was determined to be Ca_4.91_Cd_0.09_(PO_4_)_3_(OH), as calculated using Equation (8). For the HA15Cd sample, the degree of Cd^2+^ substitution at calcium sites was estimated to be *x* = 0.52 after only 3 h of contact with 15 mgCd/L aqueous solution. The refinement results indicate a progressive decrease in crystallinity with increasing cadmium content: from 57.7% in carbonated hydroxyapatite (cHA), to 50.13% in HA5Cd, and 47.42% in HA15Cd. This decline is attributed to increased lattice distortion and structural disorder induced by the incorporation of Cd^2+^ ions. The observed expansion in unit cell parameters and unit cell volume reflects the accommodation of cadmium ions within the lattice, which differs from calcium in both ionic radius and coordination behavior [58]. Additionally, the structural density and molecular weight of the unit cell increase significantly—from 3.20 to 3.58 g/cm^3^ and from 1016.9 to 1142.8 g/mol, respectively—due to the higher atomic mass of cadmium. These findings underscore the pronounced impact of Cd^2+^ adsorption on the crystal structure and properties of hydroxyapatite.

## 3. Discussion

Carbonated hydroxyapatite with a substitution degree of *y* = 0.13–0.17, as determined from FTIR analysis and structural refinement using the Rietveld method, was synthesized with granules ranging from 2 to 27 μm and an average size of 6.774 ± 4.657 μm. To simulate the characteristics of industrial wastewater, the concentrations of heavy metal solutions (Cd^2+^) used in the adsorption tests were set to be 5–15-fold higher than the total allowable concentration of heavy metals (lead, cadmium, chromium, copper, nickel, zinc, mercury) in surface water sources, according to regulations. However, in drinking water, the concentration of cadmium ions must be 0 mg/L. In the case of nitrate concentrations, the test solutions exceeded the permissible limit of 25 mg/L by up to 12 times (Romanian Norm NTPA-001-2002). The material exhibited a porous morphology, as observed in SEM images, consisting of nanometric particles with an average size of 13.529 ± 6.429 nm (TEM images), and a relatively low crystallinity of approximately 57%. This material was specifically synthesized to enhance its capacity for nitrate and Cd retention. The comparative analysis of nitrate (NO_3_^−^) adsorption capacities in a dynamic adsorption routine, and under single-ion and competitive conditions revealed significant differences. Under single-ion conditions, using KNO_3_ solutions (pH~7) with initial concentrations of 100 mg NO_3_^−^/L, the hydroxyapatite (HA) samples exhibited adsorption capacities of 18.7 mg NO_3_^−^/g for HA100N, 9.3 mg NO_3_^−^/g for HA50N, and 2.2 mg NO_3_^−^/g for HA10N (10 mg NO_3_^−^/L). In contrast, under competitive conditions—where HA samples were exposed to cadmium nitrate solutions in 2% HNO_3_ (pH < 3)—the NO_3_^−^ adsorption capacities were notably lower for similar or higher initial concentrations. Specifically, HA5Cd (101.6 mg NO_3_^−^/L) adsorbed 9 mg NO_3_^−^/g, HA10Cd (with 211 mg NO_3_^−^/L initial concentration) adsorbed 24 mg NO_3_^−^/g, and HA15Cd (316.6 mg NO_3_^−^/L) achieved 28.5 mg NO_3_^−^/g (Table 2). Notably, when comparing HA5Cd and HA100N—both exposed to similar initial nitrate concentrations (~100 mg NO_3_^−^/L)—the nitrate removal capacity under single-ion conditions was approximately twice as high, highlighting the inhibitory effect of the high acidity of the cadmium nitrate solution with 2% HNO_3_ and not of the cadmium ions presence.

**Figure 12:**
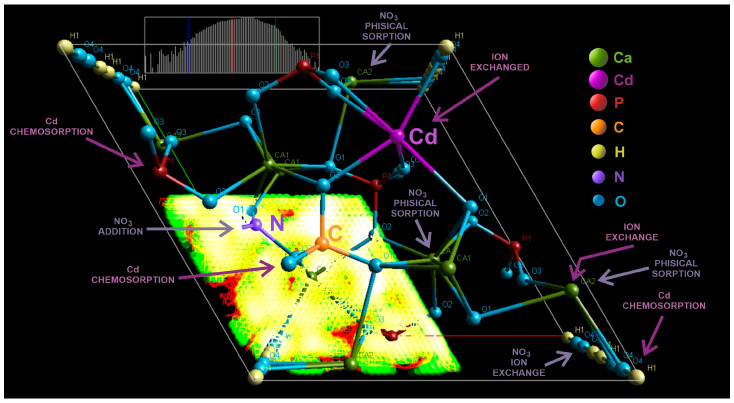
3D graphic of the cHA crystal unit cell (Fourier map), generated after XRD Rietveld structural refinement, showing the atoms positions, probable substitutions Ca (II) →Cd^2+^, PO_4_^3−^→CO_3_^2−^ and addition of NO_3_^−^, and potential physical and chemical sorption of Cd and NO_3_^−^.

Following the interaction of carbonated hydroxyapatite (cHA) powder with concentrated KNO_3_ and Cd (NO_3_)_2_ solutions (acidified with 2% HNO_3_), and based on FTIR, EDS, and XRD analyses, both NO_3_^−^ and Cd^2+^ ions were positioned in the cHA lattice. The unit cell model of cHA, refined by the Rietveld method (Figure 12), illustrates the potential mechanisms of ions adsorption. Nitrate adsorption on cHA primarily involves physical adsorption and ion exchange and secondly the addition, processes occurrence being up to pH evolution and contact time. Physical sorption—driven at pH 6–7 by van der Waals forces and diffusion gradients—dominates during short contact times (0.5–1 h), as evidenced by removal efficiencies exceeding 80% for 1–2 g of cHA after 60 min, for the powder contact with different concentrations of KNO_3_ [36]. Moreover, electrostatic interactions may also contribute under acidic conditions (pH 0.5–3, as in Cd(NO_3_)_2_ + HNO_3_) by the side of physio sorption, where the protonated cHA surface becomes positively charged, and marginal hydroxyl groups are exchanged with NO_3_^−^. This is accompanied by a pH increase from ~0.5–3 to 4.5–5 (Equation (1)) and from 5.5–6 to ~7 (Equation (2)), supporting the following reactions:

(1) $$HA-Ca-{OH}^{2+}+{NO}_{3}^{-}\to HA-Ca-{NO}_{3}+{HO}^{-}$$

(2) $$HA-Ca-OH+{NO}_{3}^{-}\to HA-Ca-{NO}_{3}+{HO}^{-}$$

FTIR analysis confirmed no phosphate substitution by NO_3_^−^, as indicated by the increased intensity of P–O vibrational bands after nitrate exposure. However, B-type CO_3_^2−^ substitution (replacing PO_4_^3−^ during synthesis) introduces local structural distortions and charge imbalance due to shorter C–O bond lengths (~1.28 Å vs. P–O ~1.51 Å), planar geometry, and lower charge of CO_3_^2−^. These effects, often compensated by calcium vacancies, facilitate NO_3_^−^ incorporation near carbonate sites—not by substitution, but by addition for charge-balancing interactions [27]. The presence of CO_3_^2−^ thus enhances NO_3_^−^ ion addition, due to its structural and electronic similarity to NO_3_^−^. FTIR confirmed this by the increased intensity and red shift of C–O vibrational bands (1420 → 1380 cm^−1^ and 875 → 826 cm^−1^), consistent with N–O vibrations. In the case of Cd(NO_3_)_2_ + HNO_3_ exposure, NO_3_^−^ was also retained via surface complexation [59]. Rietveld refinement revealed significant lattice distortions induced by anion accumulation at the particle surface, increasing structural disorder.

When the Cd(NO_3_)_2_ + HNO_3_ solution was brought into contact with the cHA powder, Cd adsorption occurred through mainly two mechanisms—chemisorption and ion exchange—simultaneously with the adsorption of NO_3_^−^ ions. The HA lattice contains two Ca sites with ion exchange potential: Ca (I), coordinated by nine phosphate oxygens, and Ca (II), coordinated by seven phosphate and one hydroxyl oxygen, the latter being more susceptible to substitution. In case of Cd adsorption, the dominant mechanism is the replacement of Ca^2+^ (Cd^2+^ ↔ Ca^2+^ ion exchange) within the cHA lattice, favored due to the isovalent nature of Cd^2+^ and Ca^2+^, along with Cd’s smaller covalent radius (148 vs. 174 pm), preference for lower coordination numbers (6–8), higher electronegativity (1.69 vs. 1.00), stronger affinity for oxygen (Equation (3)) and high acidity environment [54]. This substitution is thermodynamically favorable and supported by the formation of strong Cd–O bonds (~2.1 Å) [14]:

(3) $${Ca}_{10}{{(PO}_{4})}_{6}{\left(OH\right)}_{2}+x{Cd}^{2+}\to {Ca}_{10-x}{{{Cd}_{x}(PO}_{4})}_{6}{\left(OH\right)}_{2}+x{Ca}^{2+}$$

The amplified O–H stretching and bending FTIR bands at 3670, 1643, and 631 cm^−1^ compared with the sample before exposer also suggest Cd(OH)_2_ formation on the surface of cHA particles at elevated pH.

At high Cd^2+^ concentrations or high pH of contact solution, Cd-containing phases such as Cd(OH)_2_, Cd_3_(PO_4_)_2_, or even amorphous Cd-phosphates occurred through chemisorption as secondary sink on the cHA surface, involving electrostatic attraction to negatively charged surface groups (PO_4_^3−^, CO_3_^2−^, OH^−^). Furthermore, after Rietveld refinement, solid solutions of the type Ca_10-x_Cd_x_(PO_4_)_6_(OH)_2_ (*x* = 0.09–0.52) were detected after just 3 h of exposure to 5 and 15 mg Cd/L solutions, although unit cell parameters remained largely unchanged.

Nevertheless, the implementation of strategies for the simultaneous removal of heavy metals and nitrates using hydroxyapatite must overcome several challenges, including the material’s pH sensitivity to acidic environment and, more critically, the efficient separation of nanoparticle adsorbents following the wastewater filtration process—an issue commonly encountered in the application of nanomaterials for water treatment.

## 4. Materials and Methods

### 4.1. Carbonated Hydroxyapatite Synthesis from Eggshell

Ordinary hen eggshells sourced from Crevedia, Romania, were employed as a natural calcium precursor. To eliminate organic matter, the shells were boiled for 4 h in distilled water containing 3% hydrogen peroxide (H_2_O_2_), followed by thorough rinsing, drying at 100 °C for 1 h in an electric oven, and manual grinding for 15 min using an agate mortar and pestle [16]. The resulting powder was then calcined in a corundum crucible using a programmable electric furnace (Nabertherm, Lilienthal, Germany), with a controlled heating rate of 10 °C/min up to 670 °C and subsequently to 900 °C, each held for 3 h. The material was allowed to cool gradually to room temperature (Equation (4)) [14]. The obtained calcium oxide (CaO) powder (10 g) was dispersed in 200 mL of distilled water and magnetically stirred for 30 min to yield calcium hydroxide (Ca(OH)_2_) (Equation (5)). A 38.5% aqueous solution of dibasic ammonium phosphate ((NH_4_)_2_HPO_4_, Merck KGaA, Darmstadt, Germany) was then added dropwise at a constant rate of 2 mL/min under continuous stirring. The stoichiometric amount was calculated to achieve a Ca/P molar ratio of 1.67, maintaining the pH above 11 throughout the reaction (Equation (6)).

(4) $$CaCO_{3}\to CaO+CO_{2}$$

(5) $$CaO+H_{2}O\to Ca(OH{)}_{2}$$

(6) $$10Ca(OH{)}_{2}+6(NH_{4}{)}_{2}H(PO_{4})\to {Ca}_{10}(PO_{4}{)}_{6}(OH{)}_{2}+12(NH_{4})(OH)$$

The hydroxyapatite precipitate was maturated for 48 h at room temperature (22 °C), then 400 mL of distilled water was added under mechanical stirring. The separation of hydroxyapatite from the reaction products (ammonium hydroxide) was achieved by placing the entire amount of precipitate in the oven at 100 °C for 5 h, until complete drying and the elimination of ammonia by volatilization. The complete elimination of ammonium hydroxide is confirmed by mixing 0.1 g of powder with 10 mL of distilled water and measuring a neutral pH. If the pH is still basic, the dried powder will be resolubilized and the suspension re-dried. The resulting powder was grounded in an agate mortar and pestle for 5 min.

### 4.2. Methods

The following equipment have been used for specimen compositional, morpho-structural and functional characterization:

X-ray diffraction (XRD) performed using Bruker D8 Advance Diffractometer, operating in the Bragg–Brentano configuration with Cu-Kα (λ = 1.5406 Å), recorded between 10° < 2θ < 80° with a scan speed of 0.5°/min, a step of 0.02° and matched with the corresponding data from ICDD (Powder Diffraction Files PDF 5+). The crystallinity, unit cell parameters (*a*, *b*, *c*, *α*, *β*, *γ*), volume and average crystallite size of cHA powder samples have been determined using the Rietveld structural refinement method, based on all X-ray diffraction peaks profiles in the pattern, using HighScore Plus v3.0 and the pseudo-Voigt function for profile fitting [60]. For the calculation of the partial substitution degree of CO_3_^2−^ for PO_4_^3−^ (*y*), the refined structural data located under the “Atom Coordinates” tab were used [61]. Specifically, the phosphorus site occupancy (P_occ_) was applied in the following equation (Equation (7)) and similarly, the substitution degree of Ca_2_ sites in the hydroxyapatite lattice by Cd via ionic exchange can be quantitatively determined using Equation (8):

(7) $$y=3\times (1-P_{occ})$$

(8) $$x=5\times (1-{Ca}_{occ})$$

where *y* and *x* is C or Cd partial substitution degree, Ca_occ_ and P_occ_ represent calcium or phosphorus site occupancy.

Fourier Transform Infra-Red Spectroscopy (FTIR) spectra were recorded in the range of wave number 4000–500 cm^−1^, increments of 1.928 cm^−1^, using a JASCO FTIR-4100 spectrometer, at room temperature at a resolution of 4 cm^−1^, have been scanned 32 samples between 4000 and 440 cm^−1^, on a KBr pellets. A relative substitution index (semi-quantitative calculation) of the CO_3_^2−^ content that replaced the PO_4_^3−^ positions by reporting the intensities of the characteristic peaks from the wave numbers~1415–1460 cm^−1^ (ν_3_ antisymmetric stretching) R1 and R2 for the rate between~870 cm^−1^ (ν_2_ out-of-plane bending) of CO_3_^2−^ for PO_4_^3−^ ~1030–1100 cm^−1^ (ν_3_ antisymmetric stretching) (Equations (9) and (10)) [49,50].

(9) $$C/P=A_{{{CO}_{3}}^{\nu_{2}},\nu_{3}}/A_{{PO}_{4}v3}$$

(10) $$R_{avg}=\frac{R1+R2}{2}$$

where ACO_3_ν_2_ν_3_ is the peak height for CO_3_^2−^ absorption band at ~1415–1460 and ~870 cm^−1^; APO_4_ν_3_ is the peak height for PO_4_^3−^ absorption band at ~1030–1100 cm^−1^.

A Hitachi SU-70 Shottky Field Emission Scanning Electron Microscope (SEM) (Tokyo, Japan), equipped with an EDS probe (Electron Dispersive Spectroscopy) detector, was used in powder sample structure characterization at an acceleration voltage of 25 KV and point-to-point resolution of 1.2 nm.

The hydroxyapatite nanoparticle morphology and crystallinity details have been performed with a TECNAI F 30G2 SWIN Transmission Electron Microscope (TEM) (Thermo Fisher, Eindhoven, The Netherlands), with 300 kV accelerating transmission with a Shottky electron emission, HRTEM point and line resolution of 2 Å and 1.02 Å, respectively, a magnification range of 60x – 1 Mx, and a minimum diffraction angle of ±120°.

Particle size distribution and specific surface area of the cHA powder have been determined using a Laser Particle Sizer (Fritch Analisette 22, Idar-Oberstein, Germany), with a Mie dispersion theory for light scattering in the wet mode. The measured data were evaluated using ‘MaS control’ 3x software.

The sorption capacity (Q_e_, mg/g) and removal efficiency (E, %) of cHA samples for nitrate (NO_3_^−^) have been determined using the dynamic method, in a single-ion system described below: into a 250 mL glass conical flask with stopper, over each amount of cHA powder which varied between 0.3 and 2 g, 50 mL of deionized water was added and mixed for half hour. Afterwards, 100 mL of each of the three concentrations of nitrate solution (10 mg/L, 50 mg/L and 100 mg/L NO_3_^−^) was put in. The three testing solutions were prepared by dilution from a standard nitrate solution (Sigma Aldrich, Steinheilm, Germany, 1000 mg/L ± 4 mg/L). The cHA powder samples were exposed for 0.5, 1, 1.5, and 3 h to mentioned nitrate solutions by magnetic stirring in order to create a dynamic routine of adsorption [6,62]. All experiments were performed at ambient temperature (22 ± 2 °C) and 50 ± 2 °C. After continuous magnetic stirring at approximately 400 rpm for a predetermined contact time interval, the supernatant liquid was separated from the powder by gravitational filtration (filter paper 650d). Thus, the obtained supernatant solution samples were prepared for Jenway 6400 spectrophotometer testing (Keison Products, Chelmsford, UK) to determine the absorbance that according with the Lambert-Beer law, is proportional with the concentration of the remaining nitrates.

The adsorption characteristics of cHA powder samples towards Cd (II) ions was performed using a similar protocol as in the case of nitrate by the batch equilibrium method and dynamic regime for adsorption processes. Therefore, between 0.3 and 2 g of the cHA sample was added to aqueous stock solutions of cadmium nitrate with concentrations ranging from 5 to 15 mg Cd/L + 2% HNO_3_. The required concentrations (5, 10, 15 mg/L) were prepared from cadmium standard solution for AAS (Sigma Aldrich, Steinheilm, Germany, 1000 mg/L ± 4 mg/L, 2% HNO_3_) by successive dilutions in deionized water. The pH of solutions was measured before and after contact with sorbent powder, but without any change. Subsequently, the suspensions were magnetic stirred in order to create the dynamic conditions for adsorption process, at 400 rpm for 0.5, 1, 1.5, and 3 h to reach ionic equilibrium. The filter papers (450d) were used for separation of retentate material, and the filtrates were analyzed by the flame method at atomic absorption spectrometer (Analytik Jena GmbH—contrAA700, Jena, Germany) to established the Cd (II) remanent concentration into the supernatant liquids [11,38].

The removal efficiency E (%), for Cd and NO_3_ ions, and the amount of metal-ion adsorbed at equilibrium per unit mass of the investigated adsorbent, adsorption capacity (Q_e_, mgg^−1^), were calculated by using the following Equations (11) and (12) [51,62]:

(11) $$\%\;E=\frac{{(C}_{0}-C_{e})\times 100}{C_{0}}$$

(12) $$Q_{e}=\frac{{(C}_{0}-C_{e})\times V}{m}$$

where C_0_ (mg L^−1^) is the initial NO_3_ and Cd^2+^ concentration, C_e_ (mg/ L) is the equilibrium concentration of NO_3_ and Cd^2+^ ions in aqueous solution, V (L) is the volume of solution, m (g) is the mass of the cHA sorbent powder and Q_e_ (mg/L) is the calculated NO_3_ and Cd^2+^ adsorption amount onto cHA powder.

The separated powder samples were dried in an electric oven at 60 °C for 3 h in order to be tested. The compositional and structural changes in hydroxyapatite crystals after NO^3−^ and Cd^2+^ concentrate solution exposure were determined by FTIR, EDS and X-ray diffraction analysis and Rietveld structural refinement.

## 5. Conclusions

Carbonated hydroxyapatite (cHA) nanopowder with B-type carbonate substitutions (*y* ≈ 0.171) was synthesized from eggshell waste using minimal grinding and without thermal treatment. The resulting material demonstrated effective dual-function adsorption capabilities for both nitrate (NO_3_^−^) and cadmium (Cd^2+^) ions. Despite its relatively low specific surface area (7.568 m^2^/g), the cHA achieved over 90% nitrate removal in single-ion systems after 3 h. Under acidic and competitive conditions due to surface protonation, adsorption equilibrium was reached within 3 h for cadmium removal that exceeded 99%, with an adsorption capacity surpassing 3 mg/g, while nitrate removal efficiency increased to approximately55%. Cadmium ions were retained via a combination of physisorption, chemisorption on active surface sites, and ion exchange, leading to solid solution formation (*x* = 0.09–0.52). The results suggest that nitrate retention is closely linked to the degree of carbonate incorporation in the cHA lattice. These findings designate carbonated hydroxyapatite powder as an excellent adsorbent substrate for cadmium and nitrates, under both single-ion conditions and anion–cation competitive scenarios, in both strongly and weakly acidic environments.

## Figures and Tables

**Table 1:** Unit cell parameters for reference patterns PDF00-064-0738, PDF01-075-3728, and cHA powder samples determined by the structural refinement Rietveld method.

Unit Cell Parameters Calculated After Rietveld Structural Refinement		PDF 00-064-0738 Ca_10_(PO_4_)_6_(OH)_2_ Hexagonal P63/m Ref. Pattern	PDF 01-075-3728 Ca_5_(PO_4_)_2.829_(CO_3_)_0.26_(OH)_2.6_ Hexagonal P63/m Ref. Pattern	cHA		cHA	cHA
				59.2% Ca_10_(PO_4_)_6_(OH)_2_ Hexagonal, P63/m PDF 00-064-0738	40.8% Ca_5_(PO_4_)_2.829_(CO_3_)_0.26_(OH)_1.59_ Hexagonal, P63/m PDF 01-075-3728	100% Ca_10_(PO_4_)_6_(OH)_2_ Hexagonal, P63/m PDF 00-064-0738	100% Ca_5_(PO_4_)_2.829_(CO_3_)_0.26_(OH)_1.59_ Hexagonal, P63/m PDF 01-075-3728
a (Å) (deviation)		9.4210	9.480	9.4157 (0.016)	10.4640 (0.018)	9.4155 (0.002)	9.4183 (0.002)
b (Å) (deviation)		9.4210	9.480	9.4157 (0.016)	10.4640 (0.018)	9.4155 (0.002)	9.4183 (0.002)
c (Å) (deviation)		6.8800	6.885	6.8765 (0.018)	6.9184 (0.032)	6.8752 (0.001)	6.8771 (0.0017)
V (Å^3^)		528.83 (Z:1)	535.92 (Z:2)	527.97	656.058	527.84	528.31
α (°)		90	90	90	90	90	90
β (°)		90	90	90	90	90	90
γ (°)		120	120	120	120	120	120
Crystallinity (%)		-	-	57.47		58.28	57.7
Average crystallite size (nm)		-	-	13.01 ± 1.958	1.45 ± 0.055	12.94 ± 1.458	9.17 ± 1.650
Micro-strain (%)		-	-	0.136 ± 0.059		0.142 ± 0.042	0.165 ± 0.00001
Agreed indices	R_exp_	-	-	11.38		11.33	9.25
	R_p_	-	-	7.08		9.31	8.70
	GOF	-	-	0.682		1.190	1.639

**Table 2:** The powder sample denomination and initial concentrations of aqueous solutions related to Cd and NO_3_.

Powder Sample Denomination (3 h Contact Time, at 25 °C)	Initial Concentration of Cd and NO_3_ in Cd (NO_3_)_2_aquous Solution		Initial Concentration of NO_3_ in KNO_3_aquous Solution
	Cd^2+^	NO_3_	
cHA	-	-	-
HA10N	-	-	10 mgNO_3_/L
HA50N	-	-	50 mgNO_3_/L
HA100N	-	-	100 mgNO_3_/L
HA5Cd	5 mgCd/L	105.5 mgNO_3_/L	-
HA10Cd	10 mgCd/L	211 mgNO_3_/L	-
HA15Cd	15 mgCd/L	316.6 mgNO_3_/L	-

**Table 3:** Unit cell parameters for the cHA powder sample and HA10N, HA100N, HA5Cd and HA15Cd determined by the Rietveld structural refinement method.

Unit Cell Parameters Under Rietveld Refinement		cHA	HA5N		HA100N		HA5Cd	HA15Cd
		100% Ca_5_(PO_4_)_2.829_(CO_3_)_0.26_ (OH)_1.59_ Hexagonal, P63/m PDF 01-075-3728	83.5% Ca_10_(PO_4_)_6_ (OH)_2_ Hexagonal P63/m PDF 00-064-0738	16.5% Ca_5_(PO_4_)_2.829_(CO_3_)_0.26_ (OH)_1.59_ Hexagonal, P63/m PDF 01-075-3728	61.6% Ca_10_(PO_4_)_6_ (OH)_2_ Hexagonal P63/m PDF 00-064-0738	38.4% Ca_5_(PO_4_)_2.829_(CO_3_)_0.26_(OH)_1.59_ Hexagonal, P63/m PDF 01-075-0425	100% Ca_4.94_Cd_0.06_ (PO_4_)_3_(OH) Hexagonal, P63/m PDF04-017-8041	100% Ca_4.94_Cd_0.97_ (PO_4_)_3_(OH) Hexagonal, P63/m PDF01-075-0425
a (Å) (deviation)		9.4183 (0.002)	9.394 (0.0021)	9.418 (0.0043)	9.4032 (0.001)	9.4772 (0.0000)	9.4108 (0.0012)	9.4230 (0.0010)
b (Å) (deviation)		9.4183 (0.002)	9.394 (0.0021)	9.418 (0.0043)	9.4032 (0.001)	9.4772 (0.0000)	9.4108 (0.0012)	9.4230 (0.0010)
c (Å) (deviation)		6.8771 (0.0017)	6.9022 (0.002)	6.8929 (0.0058)	6.8923 (0.0011)	6.8886 (0.0000)	6.888 (0.0009)	6.895 (0.0009)
V (Å^3^)		528.31	527.53	535.01	527.77	536.84	528.33	530.26
Structural density (g/cm^3^) Unit cell atomic mass (g/mol)		3.21 1020.3	3.15 1000.7	3.16 1020.3	3.15 1000.7	3.17 1020.3	3.20 1016.9	3.58 1142.8
α (°) = β (°)		90	90	90	90	90	90	90
γ (°)		120	120	120	120	120	120	120
Crystallinity (%)		57.7	50.49		48.14		50.13	47.42
Average crystallite size (nm)		9.17 ± 1.650	16.467 ± 2.482	10.253 ± 1.559	19.92267 ± 2.844	12.447 ± 0.974	16.457 ± 1.446	15.991 ± 2.046
Micro-strain (%)		0.165 ± 0.00001	0.624 ± 0.325		0.750 ± 0.391		0.565 ± 0.234	0.985 ± 0.273
Agreed indices	R_exp_	9.25	11.33		12.16		11.96	11.71
	R_p_	8.70	8.46		9.58		9.63	9.86
	GOF	1.639	1.02		1.08		1.31	1.38

## Data Availability

Data is contained within the article and Supplementary Materials.

## Supplementary Materials

The following supporting information can be downloaded at: https://www.mdpi.com/article/10.3390/ijms26167766/s1.
