# Supplementary material for: Sustainable Synthesis and Dual-Function Sorption of Carbonated Hydroxyapatite for Cadmium and Nitrate Removal
Source: Int J Mol Sci. 2025 Aug 11;26(16):7766. doi: 10.3390/ijms26167766 (PMC12386647; doi:10.3390/ijms26167766)
Supplement: Supplementary file 1 [file ijms-26-07766-s001.zip › ijms-3794616-supplementary.pdf]

Supplementary Materials

Table S1. *Statistical data of the three samples of cHA powder measured by Laser Diffraction Analyzer*

Statistic data	cHA POWDER SAMPLE		
	362	363	364
Arithmetic Mean Diameter μm	0.954	0.444	0.461
Geometric Mean Diameter μm	0.865	0.271	0.281
Quadr. Sq. Mean Diameter μm	1.517	0.628	0.653
Harmonic Mean Diameter μm	1.263	0.185	0.191
Variance μm^2	0.277	0.200	0.216
Mean Square Deviation μm	5.757	0.447	0.465
Average Deviation μm	0.273	0.384	0.396
Coefficient of variance %	33.347	100	100.9
Skewness	0.988	2.770	2.622
Curtosis	0.778	0.644	0.655
Span	0.867	5.786	5.699
Uniformity	0.297	1.787	1.760
Spec. Surface Area (cm^2/cm^3)	80,117.447	323,636.751	313,693.996

Table S2. *The absorbance band amplitude for different bound vibrations corresponding*

Sample	Bond vibration	FTIR absorption band	
		Y (arbitrary unit)	X (cm^{-1})
HA100N	H-O (cHA)	0.835	3600-3000 Centred at 3419
HA50N		0.586	
HA10N		0.457	
HA15Cd		0.470	
HA10Cd		0.530	
HA5Cd		0.438	
cHA		0.217	
HA100N	C—O in $\nu_3(CO_3^{2-})/\ \nu_2$ trigonal	1418/872	1.1754/0.1052
HA50N		1419.5/874	1.0539/0.1827
HA10N		1420/875	0.8477/0.2554
HA15Cd		1420/874.1	0.2484/0.3268
HA10Cd		1420/874.4	0.2621/0.2954
HA5Cd		1421/874.4	0.2855/0.2699
cHA		1420/875	0.3518/0.122
HA15Cd	P—O in ν_3PO_4 tetrahedra	1043	3.818
HA10Cd		1042	3.686
HA5Cd		1042	2.794
cHA		1039	1.569
